# Where are emotions in words? Functional localization of valence effects in visual word recognition

**DOI:** 10.3389/fpsyg.2014.01105

**Published:** 2014-09-30

**Authors:** Marina Palazova

**Affiliations:** General Psychology and Neurocognitive Psychology, International Psychoanalytic University BerlinBerlin, Germany

**Keywords:** emotions, visual word recognition, linguistic representation, emotional valence, lexical access

Although emotional processing in words became a strong focus of research recently, less attention was given to the question of functional localization of emotion effects in the stream of visual word recognition directly. Here, the impact of emotional connotation of words on different processing stages of reading (pre-lexical, lexical, or semantic) is investigated. Or put alternatively: How is emotional valence represented within the linguistic representational system?

From a psycholinguistic perspective there are at least two types of linguistic representations which are central to visual word recognition. These are lexical and semantic representations. It is a challenging endeavor to define the term lexical: Whether low-level lexical representations (pure orthographic processing or the visual word form) should be differentiated from higher-level lexical representations (denoting, e.g., word frequency), is for example an open issue. Furthermore, orthographic processing may comprise sublexical processing on the level of letters and syllables, and lexical processing on the level of the complete word form. The term semantic commonly refers to the meaning of words, presumed as internally represented concepts made of smaller elements of meaning organized by semantic similarity.

In psycholinguistics separate lexical and semantic representations are presumed. Accordingly, most models of visual word recognition assume that lexical representations are retrieved (lexical access) after basic low-level visual perception of line forms and colors, which then culminate in activation of semantic knowledge. Models of word recognition differ with respect to their assumptions about discreteness of the processing stages and to mechanisms of accessing the lexical and semantic representations. While early models of visual word recognition postulated discrete processing stages (e.g., Forster, 1976) more recent computational approaches (e.g., Coltheart et al., 2001) assume interactive processing stages organized in a cascaded manner. To my knowledge, there is no single visual word recognition model though simulating both lexical and semantic effects. Thus, emotional valence seems an interesting factor, since there is an ongoing debate about whether it should be understood as a lexical or as a semantic factor. Insights into the linguistic representations related to emotional valence would deliver important implications for visual word recognition models in general.

For comparison of the time course of emotion effects and visual word recognition the prominent event-related potential (ERP) components in visual word processing should first be considered irrespective of emotion. Higher-level lexical representation effects (e.g., of word frequency) are observed already 100-ms post-stimulus. Since word frequency is broadly accepted to be a lexical factor, such modulations imply that lexical access is underway already starting in the time course of the P1 (Assadollahi and Pulvermüller, 2003; Hauk et al., 2006; Palazova et al., 2011). Earliest effects reported for semantic factors start at 160 ms (Hauk et al., 2012). Nevertheless, a more conservative view on word recognition postulates a timeline of 150 ms for pre-lexical and low-level lexical processing, at 250 ms for lexical and at 400 ms for semantic access (e.g., Grainger and Holcomb, 2009). Such results have some very important implications for the understanding of word recognition processes: (i) there seems to be a certain variability of onsets of separate linguistic processing stages in time, and (ii) the early effects may also indicate feedback mechanisms even on sublexical/low-level lexical processing stages (Carreiras et al., 2014). A current proposal is pointing to a possible key role of the ventral occipitotemporal cortex regarding feedback mechanisms in reading (Price and Devlin, 2011). Most models of word recognition, however, assume at least in very early processing stages a feedforward mechanism without any feedback from high-level to very early processing stages.

Dimensional models of emotion have a long tradition in psychology and are among the most influential theories of emotion processing. These models suggest two main dimensions that describe the emotional space – (i) emotional valence denotes whether a stimulus is being perceived and experienced as positive or negative, and (ii) arousal constitutes the intensity of the appraisal process. I will limit the article to discussion of valence effects which can be understood as the dimension that underlies the quality of emotional experience. Considering the time course of emotional valence effects three different components of the ERP were observed with words. Very early emotion effects have been observed in the time course of P1 (Bernat et al., 2001; Hofmann et al., 2009; Bayer et al., 2012) or N1 (Kissler and Herbert, 2013) presumably reflecting activation of visual cortex. Recently, also a temporal area, the left middle temporal gyrus (MTG), has been discussed as the neural source underlying emotional P1 modulations (Keuper et al., 2014). Earliest emotion effects have been observed already starting at 50 ms after stimulus onset in the C1 component, conceivably reflecting first responses in the primary visual cortex (Rellecke et al., 2011). The second eminently reported component to emotional words is the early posterior negativity (EPN), starting approximately 200 ms after stimulus onset (Kissler et al., 2007; Herbert et al., 2008; Schacht and Sommer, 2009; Palazova et al., 2011, 2013). The EPN is an augmented negativity to emotional stimuli as compared to neutral stimuli at occipito-temporal sites, which is seen to reflect attention allocation to intrinsically relevant stimuli involving an extended network of occipital, temporal, and parietal areas (Keuper et al., 2014). The late positive complex (LPC), the third emotional ERP component, has been observed from latencies of 350 ms and higher, and consists in increased centro-parietal positivity for emotional stimuli relatively to neutral ones. An LPC has often been found in studies with written words in tasks demanding higher level lexico-semantic processing (Herbert et al., 2006, 2011; Carretie et al., 2008; Kissler et al., 2009; Schacht and Sommer, 2009; Hinojosa et al., 2010).

The timing of the separate emotion components indicates impact of emotion on several word recognition stages. While the time course of very early emotion effects seems too early to reflect fully accessed word meaning, the time course of the EPN does not allow for such a clear conclusion. A comparison of the time course of the EPN and lexical and semantic stages of visual word recognition alone does not deliver much insight into the underlying functional mechanisms. According to the described time-course of the EPN in visual word recognition, both a lexical and a semantic locus would be conceivable. Considering the conservative view on word recognition the EPN would on the one hand fully coincide with lexical processing stages from 200 ms onwards, on the other hand evidence speaking for semantic access already before 200-ms post-stimulus would indicate a semantic functional locus of emotional valence effects.

## Are effects of emotions semantic in nature?

There is clear evidence in favor of a semantic locus of emotion effects. By now a differentiated picture of results has emerged for the EPN component. As mentioned, the EPN time course is not sufficient to distinguish whether emotion effects can be semantic, that is whether these effects are a consequence of retrieved semantic representations as proposed by Kissler et al. (2007). A lexical locus may also be conceivable since a component related to semantic processing as the N400 peaks later than the latency of the EPN. Furthermore, according to the conservative view lexical processing is underway coinciding with the time course of the EPN.

Another possibility to address this question is to orthogonally combine emotional valence with other factors that are either lexical or semantic and track their interactions in accordance to the additive factor method (Sternberg, 2011). Palazova et al. (2013) followed this logic and examined the time course of emotion effects within concrete and abstract words. Word concreteness is a semantic factor which refers to whether the correspondence of a mental concept in reality can be perceived by the senses or not, and has been observed to alter response times and late components in the ERP as the N400. Importantly, emotion effects interacted with concreteness within the EPN with concrete words eliciting earlier EPN than abstract words. In the same line of arguments, Palazova et al. (2011) combined orthogonally emotional valence with word frequency, a factor that is broadly accepted to be lexical of nature. In contrast to the emotion concreteness interaction, no interactions of the factors emotion and frequency were observed for the EPN. Simultaneously long lasting main effects of both factors were observed. These two studies together deliver direct evidence for a semantic functional locus of processes reflected in the EPN. That is the presumed increased attention evoking the EPN depends on retrieval of semantic meaning of the words. Considering the fact that the LPC is generally observed after the EPN and interpreted as elaborate processing of emotional connotation, the LPC can be congruously interpreted as based on the retrieved meaning of emotional words.

## Are effects of emotions lexical in nature?

The very early emotion effects in words cannot be easily explained with the semantic locus hypothesis and have generated much debate. Two hypotheses were established to explain why and how they do emerge. First, very early emotion effects have been interpreted as a marker for facilitated and accelerated lexical access of emotional compared with neutral words (Hofmann et al., 2009). The second refers to the idea that very early emotion effects can be explained by conditioned responses to word form of emotional connotation (Palazova et al., 2011); please see also Keuper et al. (2014) for a related account on very early emotion effects at the level of lexical processing.

Speeded lexical access is conceivable since response times are shorter and lexicality effects (the first ERP difference between words and pseudowords) exhibit a shorter latency to emotional than to neutral words (Kissler and Herbert, 2013). The underlying mechanisms are still elusive. Importantly, however, the idea of speeded lexical access and the conditioning hypothesis are not mutual exclusive – it could be possible that conditioned responses to emotional words account for facilitated retrieval of (sub-)lexical representations. The question, which is not answered yet, is on which level of lexical representation exactly emotion exerts its influence. First, speeded lexical access may depend on emotion as a part of the lexical representation, which in analogy to word frequency would facilitate retrieval of higher-level lexical representations. An alternative would be feedback processing from fast accessed semantic representations of words. Emotional valence may be the first retrieved semantic feature of a word (Palazova et al., 2013), and therefore may exert facilitating feedback influence on the lexical level without emotion being represented as a part of lexical representations. This alternative seems less plausible, since the arguments for a lexical locus overweigh those against it: very early emotion effects in the C1 and P1 seem too early to reflect feedback processing from semantic processing stages, and early interactions with word frequency are a direct indication of higher-level lexical representations. On the other hand, as recently shown by Keuper et al. (2014), MTG involvement in P1 effects points to lower-level lexical representation, i.e., the visual word form. The earliest observed emotion effects (starting already 50-ms post-stimulus) and variability across observed very early emotion effects (only to negative words: Hofmann et al., 2009; or to positive Palazova et al., 2011; Bayer et al., 2012; or both Keuper et al., 2014) would even indicate the sublexical level on the basis of syllables. That is, sublexical entities, e.g., prefixes may serve as conditional cues for emotional valence information. In morphologically rich languages such as German it is conceivable that some prefixes would carry some valence information in case they are more frequently related to negative or to positive than to neutral words. The exact level of linguistic representation is still an open question and would need future research.

Taken together, it can be assumed that emotional valence is a semantic feature, possibly the first semantic feature to be retrieved from semantic memory when reading words. A growing body of evidence is pointing to a second possible locus of emotion in the lexical linguistic representations. The exact level of lexical representation and the underpinning learning mechanisms are open issues. The conclusion that emotional valence impacts word recognition on multiple stages and might be both part of lexical and of semantic representations is pinpointing future challenges for models of visual word recognition, that is, first, the need for integration of models that either have a focus on lexical or on semantic processing, and second, the integration and prediction of word dimensions like emotion within such models.

### Conflict of interest statement

The author declares that the research was conducted in the absence of any commercial or financial relationships that could be construed as a potential conflict of interest.
